# A Putative Zn(II)_2_Cys_6_-Type Transcription Factor FpUme18 Is Required for Development, Conidiation, Cell Wall Integrity, Endocytosis and Full Virulence in *Fusarium pseudograminearum*

**DOI:** 10.3390/ijms241310987

**Published:** 2023-07-01

**Authors:** Yuan Zhang, Xunyu Zhuang, Jiaxing Meng, Feifei Zan, Zheran Liu, Cancan Qin, Lingjun Hao, Zhifang Wang, Limin Wang, Honglian Li, Haiyang Li, Shengli Ding

**Affiliations:** 1 College of Plant Protection, Henan Agricultural University, Zhengzhou 450046, China; 2 National Key Laboratory of Wheat and Maize Crop Science, Zhengzhou 450046, China

**Keywords:** *Fusarium pseudograminearum*, transcription factor, Zn(II)_2_Cys_6_, endocytosis

## Abstract

*Fusarium pseudograminearum* is one of the major fungal pathogens that cause *Fusarium* crown rot (FCR) worldwide and can lead to a substantially reduced grain yield and quality. Transcription factors play an important role in regulating growth and pathogenicity in plant pathogens. In this study, we identified a putative Zn(II)_2_Cys_6_ fungal-type domain-containing transcription factor and named it FpUme18. The expression of *FpUME18* was induced during the infection of wheat by *F. pseudograminearum*. The *ΔFpume18* deletion mutant showed defects in growth, conidial production, and conidial germination. In the responses to the cell wall, salt and oxidative stresses, the *ΔFpume18* mutant inhibited the rate of mycelial growth at a higher rate compared with the wild type. The staining of conidia and mycelia with lipophilic dye FM4-64 revealed a delay in endocytosis when *FpUME18* was deleted. *FpUME18* also positively regulated the expression of phospholipid-related synthesis genes. The deletion of *FpUME18* attenuated the pathogenicity of wheat coleoptiles. *FpUME18* also participated in the production of the DON toxin by regulating the expression of *TRI* genes. Collectively, FpUme18 is required for vegetative growth, conidiation, stress response, endocytosis, and full virulence in *F. pseudograminearum*.

## 1. Introduction

*Fusarium* crown rot (FCR) is one of the most destructive soil-borne diseases of wheat in many countries [1,2]. *Fusarium pseudograminearum* is one of the major fungal pathogens causing FCR [3]. *F. pseudograminearum* has a serious impact on the global production of wheat and barley. In the northern region of Australia, the yield loss caused by *F. pseudograminearum* can reach more than 10% [4]. In China, *F. pseudograminearum* was first reported in Henan in 2012 [5], and it has become one of the dominant species causing FCR and head blight in the Huanghuai wheat-growing region [6]. In Xinxiang, Henan province, the average incidence rate of FCR has reached 59%, which led to a 70.6% yield loss in severe cases [7]. Moreover, *F. pseudograminearum* could produce deoxynivalenol (DON), nivalenol (NIV) and other mycotoxins, which seriously threaten food security alongside human and livestock health [8]. After the screening of 2514 wheat cultivars which were collected from different geographical regions worldwide, only a small proportion of wheat varieties displayed a medium or high resistance to FCR [9]. In China, only 15 out of the 670 materials tested showed a stable resistance to FCR, and the proportion of susceptible materials reached 84%, including the main planting varieties of recent years [10]. Therefore, understanding the molecular mechanism of how *F. pseudograminearum* causes the disease is vital to disease prevention and control.

*F. pseudograminearum* successfully infected and colonized host tissues by producing toxins, enzymes and effectors [8]. So far, the functional characterizations of some pathogenic genes in *F. pseudograminearum* have been reported. For example, Fpkapc, a bZIP transcription factor, is critical for pathogenicity, conidiation and oxidative stress [11]. A nonribosomal peptide gene, *FpNPS9,* plays an important role in deoxynivalenol production and host infection [12]. The peroxisome proliferator FpPex11 is involved in development and pathogenicity [13]. Despite this progress, the mechanisms of pathogenesis in *F. pseudograminearum* remain largely unknown.

Gene regulation is critical in biological processes in all organisms [14]. Transcription factors play an important role in regulating gene expression [15]. Transcription factors include a zinc finger domain that binds to DNA, and these can be classified into three categories: Cys_2_His_2_ (C_2_H_2_), Cys_4_ (C_4_) and Cys_6_ (C_6_) zinc finger proteins [16]. These proteins regulate multiple cellular processes, such as cell cycle, glucose and amino acid metabolism, and stress response [16]. C6 zinc finger proteins include six cysteine residues, which combine with two zinc atoms; therefore, they are also known as Zn(II)_2_Cys_6_ (Zn_2_C_6_) proteins. The Zn(II)_2_Cys_6_ transcription factors are only found in fungi and have been shown to play significant roles in fungal development, fungicide resistance and pathogenesis [17,18,19]. For instance, *MoIRR* is associated with isoprothiolane resistance in *Magnaporthe oryzae* [20]; *BcGaaR* regulates D-galacturonic acid utilization in *Botrytis cinerea* [21]; ADA-6 regulates conidiation, sexual development, and the oxidative stress response in *Neurospora crassa* [22]; Fdb3 is essential for *F. pseudograminearum* to efficiently detoxify the predominant wheat benzoxazolinone, 6-methoxy-benzoxazolin-2-one [23].

In this study, we identified and characterized a putative Zn(II)_2_Cys_6_ transcription factor, FpUme18, in *F. pseudograminearum* using reverse genetics. The results indicated that FpUme18 is involved in hyphal growth, conidiation, stress responses, endocytosis and pathogenicity in *F. pseudograminearum*.

## 2. Results

### 2.1. Identification and Expression of FpUME18 in F. pseudograminearum

**Figure 1:**
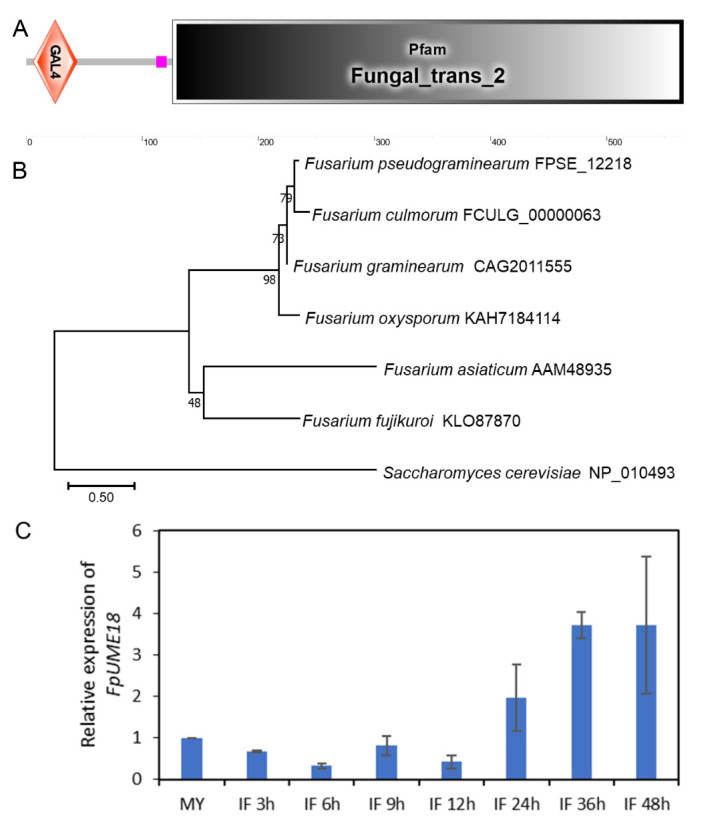
Domain, phylogenetic tree and gene expression analysis of FpUme18 in *F. pseudograminearum*. (**A**) Prediction of domains via SMART. (**B**) The evolutionary tree of FpUme18 and its orthologous proteins from *Fusarium graminearum*, *Fusarium oxysporum*, *F. culmorum*, *Fusarium asiaticum*, *Fusarium fujikuroi*, and *Saccharomyces cerevisiae* was constructed using neighbor-joining by MEGA11; bootstrap method was 1000. (**C**) The transcription level of *FpUME18* was assayed using qRT-PCR. MY, mycelia; IF 3 h to 48 h was the time course post-infection. Data are the means ± s.d. of three replicates.

Ume6 is a transcription factor in yeast with important functions in the histone deacetylase Rpd3 complex. Ume6 can regulate phospholipid metabolism, arginine metabolism, autophagy, meiosis, DNA repair and other important biological processes [24,25]. We searched for the *F. pseudograminearum* genome database with the ume6 of *Saccharomyces cerevisiae* and discovered one orthologue of *FPSE_12218* and named it *FpUME18*. *FpUME18* encodes a protein of 566 amino acids with a predicted molecular weight of 63.33 kDa. Domain analysis with SMART showed that FpUme18 had a GAL4 DNA-binding domain, with a Zn^2+^ binding site at positions 6–33, a low complexity region (pink box) at its N terminus and a fungal-specific transcription factor domain at the C terminus (Figure 1A). A phylogenetic tree of FpUme18 and its orthologous proteins in six other fungi was constructed (Figure 1B). The results showed that FpUme18 was more closely related to the orthologous protein in *Fusarium culmorum* and was consistent with known species relatedness.

To better study the function of FpUme18 during the *F. pseudograminearum* infection of wheat, the expression level of *FpUME18* during the course of infection was detected using qPCR. The transcription level of *FpUME18* significantly increased after infection for 24 h (Figure 1C), which meant that FpUme18 could play an important role in virulence.

### 2.2. Generation of FpUME18 Deletion Mutant and Complemented Transformants

To determine the biological role of *FpUME18* in *F. pseudograminearum,* we generated gene deletion mutants by replacing *FpUME18* with the hygromycin phosphotransferase resistance cassette (*HPH*) at the gene site (Figure S1A). These putative mutants were screened and confirmed using a PCR (Figure S1B) and further confirmed with genome resequencing (Figure S1C). To confirm whether phenotypic changes in the mutants were due to the deletion of *FpUME18*, we transformed the construct pYIP102::*FpUME18*::3FLAG into the *ΔFpume18* mutant, which was driven by its native promoter. Five *cFpume18* transformants with resistance to G418 were obtained, and this was confirmed using a PCR. Additionally, one of them was further confirmed by Western blot (Figure S1D,E). The *cFpume18* fully restored the defected phenotype to a wild type.

### 2.3. FpUme18 Is Involved in the Regulation of Vegetative Growth

**Figure 2:**
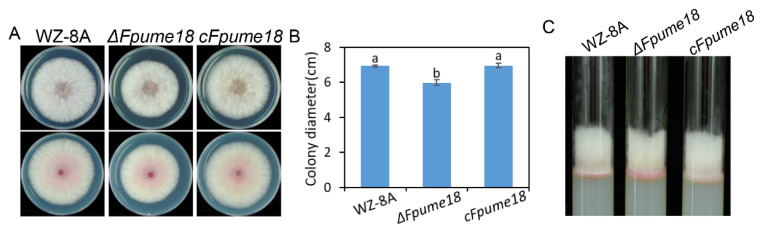
Effect of *FpUME18* on mycelium growth. (**A**) The colony morphologies of WZ-8A, deletion mutant *ΔFpume18,* and complementary strain *cFpume18* grew on a PDA plate for 3 days. (**B**) The colony diameter was determined after three days of PDA plate growth. Data are the means ± s.d. of three independent experiments with a and b above indicating significant differences at a *p*-value of 0.05. (**C**) Comparison of aerial hyphae of WZ-8A, *ΔFpume18* and *cFpume18.*

We compared the growth rates of the *ΔFpume18* mutant with WZ-8A and *cFpume18* on PDA. The colony diameter of *ΔFpume18* was significantly smaller than those of WZ-8A and *cFpume18* (Figure 2A,B). However, the phenotype for the aerial mycelia of *ΔFpume18* looked the same as those of WZ-8A and *cFpume18* (Figure 2C). The results suggested that FpUme18 could be associated with the regulation of vegetative growth in *F. pseudograminearum*.

### 2.4. FpUme18 Is Important for Conidiation and Conidia Germination

**Figure 3:**
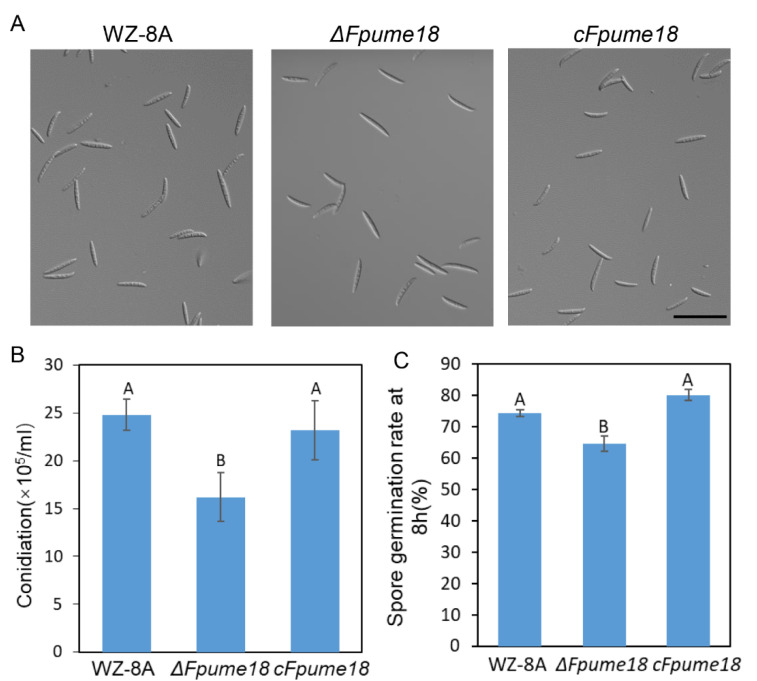
Conidiation and conidial germination assays. (**A**) The conidial phenotype produced by mycelial plugs culturing in a CMC liquid medium for 5 days. Bar = 50 μm. (**B**) Statistics analysis on conidiation of WZ-8A, *ΔFpume18* and *cFpume18.* (**C**) Conidial germination rates of WZ-8A, *ΔFpume18* and *cFpume18* strains in ddH_2_O. The above data are the means ± s.d. of three independent experiments with A and B above indicating significant differences at a *p*-value of 0.01.

We further investigated whether FpUme18 was involved in regulating conidiation and conidial germination. The results showed that the conidial production of *Δfpume18* decreased by approximately 7.1 × 10^5^/mL when compared to WZ-8A (Figure 3A,B). After 8 h of darkness treatment, the number of germinating spores of *ΔFpume18* was significantly lower than that of WZ-8A and *cFpume18* (Figure 3C), even with the elongation of the incubation time. Thus, FpUme18 was found to function in asexual reproduction and conidial germination in *F. pseudograminearum*.

### 2.5. FpUme18 Is Important for Stress Responses

**Figure 4:**
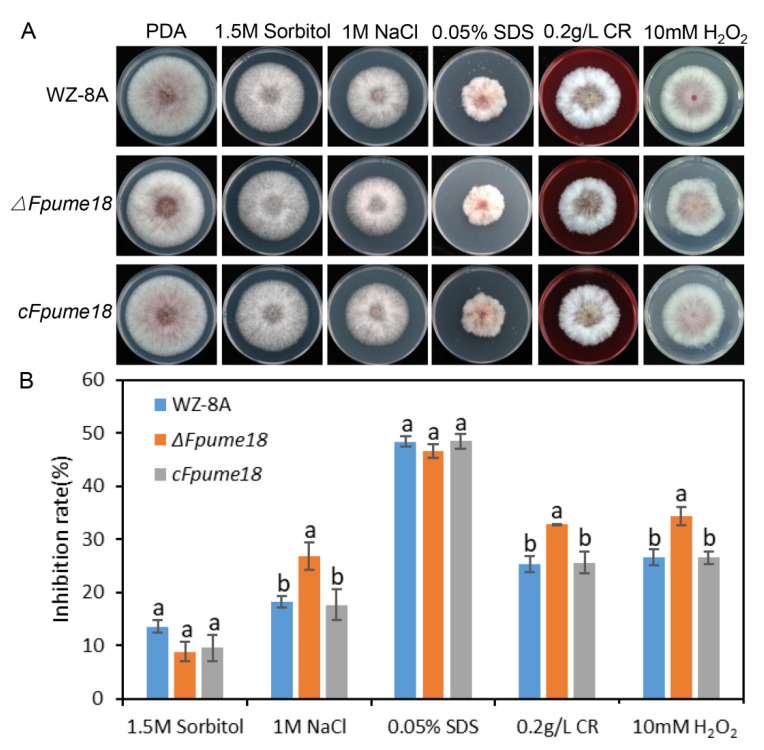
Assays on sensitivity under different stresses. (**A**) Colonies of WZ-8A, *ΔFpume18* and *cFpume18* strains on a PDA medium with and without 1.5 M Sorbitol, 1 M NaCl, 0.05% SDS, 0.2 g/L Congo Red (CR), and 10 mM H_2_O_2_. (**B**) Mycelial growth inhibition rate quantified 3 days after inoculation on a medium. Data are the means ± s.d. of three independent experiments, with a and b above indicating significant differences at a *p*-value of 0.05.

Pathogens suffer from various environmental stresses during infection. To investigate whether FpUme18 was involved in environmental stress in *F. pseudograminearum*, WZ-8A, *ΔFpume18* and *cFpUme18* were inoculated onto PDA plates with or without osmotic stress (sorbitol), salt stress (NaCl), cell membrane, cell wall stress (SDS and Congo red) and oxidative stress (H_2_O_2_) (Figure 4A). The results showed that the *ΔFpume18* mutant was more sensitive to salt stresses, cell wall stress and oxidative stress. The growth inhibition rate of the *ΔFpume18* mutant was significantly higher than WZ-8A and *cFpume18* on a medium with 1 M NaCl, 0.2 g/L CR and 10 mM H_2_O_2_ (Figure 4B). These results indicated that FpUme18 contributed to balancing salt stress, keeping the cell wall’s integrity and reactive oxygen resistance in *F. pseudograminearum* when undergoing environmental stresses.

### 2.6. FpUme18 Is Required for Normal Endocytosis

**Figure 5:**
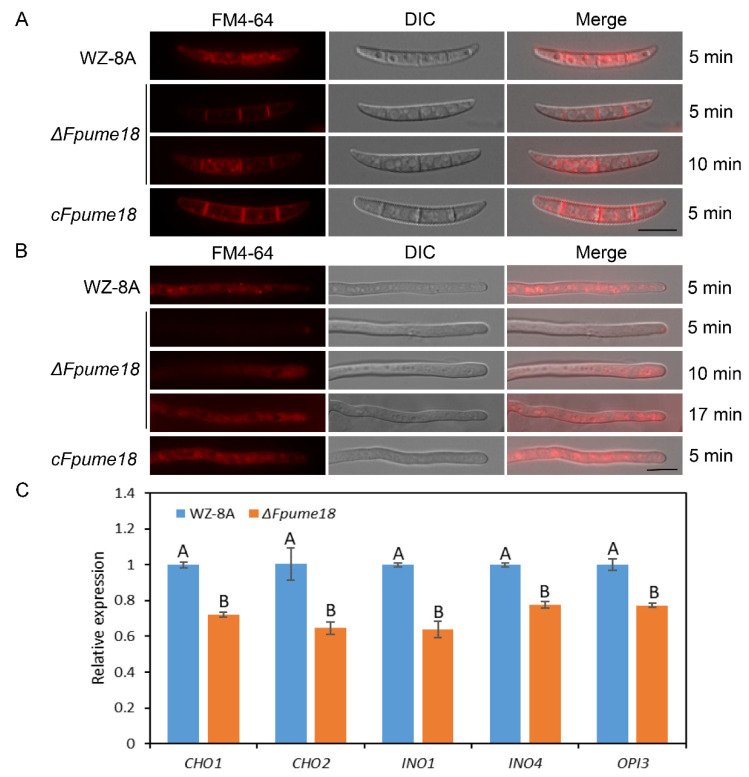
Analysis of cell endocytosis. (**A**) Staining the conidia of *ΔFpume18*, WZ-8A and *cFpume18* with FM4-64 dye. (**B**) Staining the hyphae of *ΔFpume18*, WZ-8A and *cFpume18* with FM4-64 dye. Photographs were taken at different times after exposure at 900 nm. Bar = 10 μm. (**C**) Relative expression levels of *CHO1*, *CHO2*, *INO1*, *INO4* and *OPI3* in WZ-8A and *ΔFpume18*. Data are the means ± s.d. of three replicates, with A and B above indicating significant differences at a *p*-value of 0.01.

Endocytosis transports extracellular substances into cells through the deformation of the plasma membrane, which is very important for cell function. The lipophilic dye FM4-64 could be used to track endocytosis. We incubated the conidia of *ΔFpume18*, WZ-8A and *cFpume18* with FM4-64; *ΔFpume18* showed the same degree after staining for 10 min as WZ-8A and *cFpume18* after staining for 5 min. (Figure 5A). When staining the hyphae, the red fluorescence signal of FM4-64 from the whole hyphae was detected in WZ-8A and *cFpume18* at 5 min, and the same degree of staining was observed in the *ΔFpume18* mutant at 17 min (Figure 5B). The staining results for conidia and hyphae consistently showed that the deletion of *FpUME18* delayed endocytosis in *F. pseudograminearum*, indicating that FpUme18 positively regulated vesicle trafficking.

The phospholipid is the main component of the cell membrane, and Ume6 could regulate phospholipid metabolism in yeast. The FM4-64 staining results showed that *FpUME18* could be associated with endocytosis. We detected the expression of several phospholipid-synthesis-related genes in *ΔFpume18* and WZ-8A (Figure 5C). After *FpUME18* deletion, the expression levels of *CHO1*, *CHO2*, *INO1*, *INO4* and *OPI3* were significantly downregulated during the spore germination stage. By regulating the expression of these genes, *FpUME18* could affect the synthesis of phospholipids and further regulate the normal function of the cell membrane.

### 2.7. FpUme18 Affects the Pathogenicity in F. pseudograminearum

**Figure 6:**
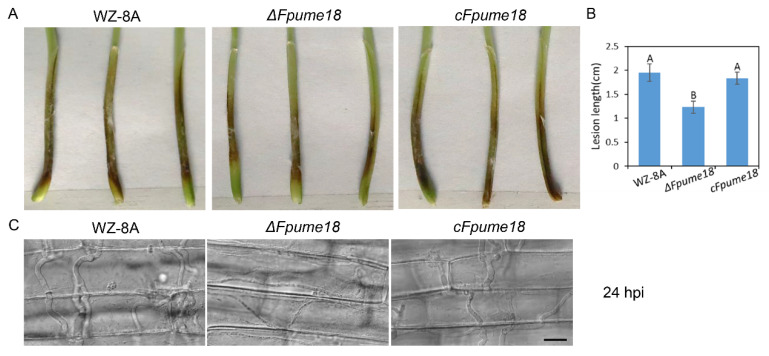
Pathogenicity assays. (**A**,**B**) The wheat coleoptiles inoculated with mycelium plugs after 3 days and lesion length. Data are the means ± s.d. of three independent experiments with A and B above indicating significant differences at a *p* value of 0.01. (**C**) Observation of wheat coleoptiles inoculated with mycelium blocks after 24 hpi. Bar = 20 μm.

In order to explore the functions of FpUme18 in the pathogenicity of *F. pseudograminearum*, we inoculated mycelium blocks of WZ-8A, *ΔFpume18* and *cFpume18* on wheat coleoptiles. After 3 days, the length of the lesion caused by *ΔFpume18* was significantly shorter than that caused by WZ-8A and *cFpume18* (Figure 6A,B). To further confirm the involvement of *FpUME18* in the infection of *F. pseudograminearum*, the infection process was observed in wheat coleoptile cells under a microscope. At 24 hpi, *ΔFpume18* was only able to penetrate a small number of hyphae into the cells, and they did not easily expand to neighboring cells. Most hyphae of WZ-8A and *cFpume18* had already entered the cells and expanded at this time (Figure 6C). These results suggest that FpUme18 was required for the full virulence of *F. pseudograminearum*.

### 2.8. FpUme18 Involved in the Production of DON Toxin in F. pseudograminearum

**Figure 7:**
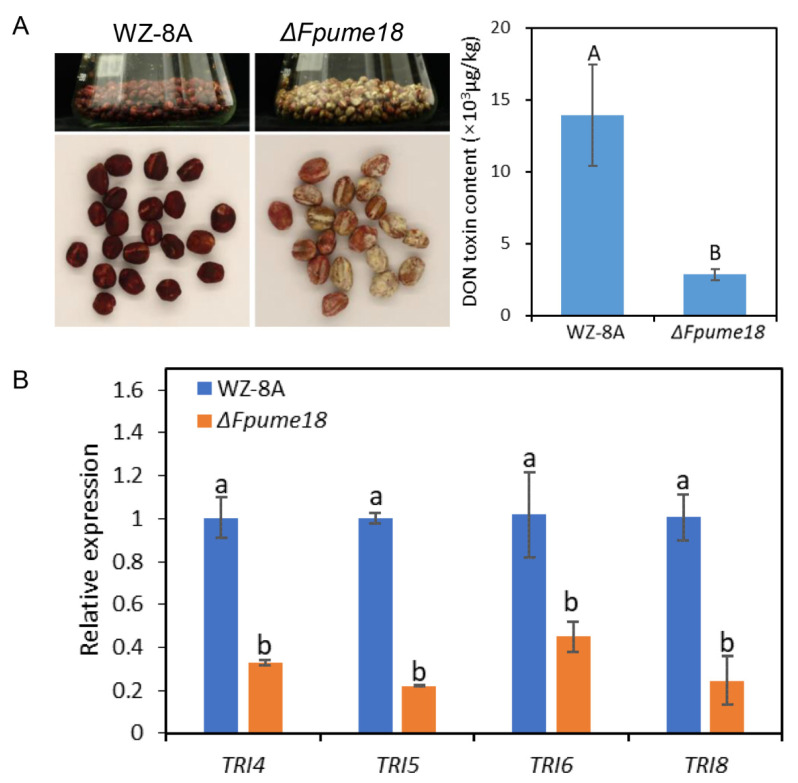
DON production and *TIR* gene expression in WZ-8A and *ΔFpume18*. (**A**) State of wheat grains after 30 days treated with WZ-8A and *ΔFpume18* and the production of DON tested with HPLC in different treatments. Data are the means ± s.d. of three experiments with A and B above indicating significant differences at a *p*-value of 0.01. (**B**) Relative expression levels of *TRI4*, *TRI5*, *TRI6* and *TRI8* in WZ-8A and *ΔFpume18* after the infection of wheat coleoptiles for 48 h. Data are the means ± s.d. of three independent experiments, with a and b above indicating significant differences at a *p*-value of 0.05.

*F. pseudograminearum* produces DON toxin while infecting wheat [8]. DON toxins can contaminate wheat stalks and grains, which is a potential threat to the health of humans and livestock. Therefore, we tested the DON production of WZ-8A and *ΔFpume18* using high-performance liquid chromatography. The results showed that the DON production of *ΔFpume18* in wheat grains was significantly lower than that of WZ-8A (Figure 7A). To further confirm this result, the expression levels of genes (*TRI* genes) for trichothecene biosynthesis were detected. The qPCR results showed that the expression levels of *TRI4*, *TRI5*, *TRI6* and *TRI8* in *ΔFpume18* significantly decreased compared with those of WZ-8A (Figure 7B). In conclusion, *FpUME18* could affect the production of DON toxin by regulating the expression of *TRI* genes.

### 2.9. FpUme18 Has Non-Transcriptional Activation Activity in the Yeast System

The prediction of the protein structure showed that FpUme18 had a GAL4 DNA-binding domain. In order to further understand how FpUme18 regulates the biological function of *F. pseudograminearum*, we characterized the transcriptional activating activity of FpUme18. The transcriptional activation activity of FpUme18 was first verified using a yeast system. Referring to Zhu et al. [26], the transformed pGBKT7-*FpUME18* yeast was grown normally on a selective dropout/–tryptophan (SD/-Trp) medium, indicating its successful fusion with the yeast strain. However, the transformed pGBKT7-*FpUME18* yeast could not grow on the selective dropout/-tryptophan-histidine-adenine + X-α-gal (SD/-Trp-His-Ade + X-α-gal) medium, indicating that it could not activate the transcription of downstream reporter genes. These results show that FpUme18 has Non-transcriptional activation activity in the yeast system (Figure S2).

## 3. Discussion

In this study, we identified a putative Zn(II)_2_Cys_6_-Type transcription factor FpUme18. FpUme18 was required for the pathogenicity of *F. pseudograminearum* on wheat and the absence of *FpUME18* significantly reduced pathogenicity, and the production of the DON toxin during infection for *F. pseudograminearum*. Additionally, FpUme18 was involved in vegetative growth, conidiation, conidial germination and stress responses. The deletion of *FpUME18* led to downregulated expressions of phospholipid-synthesis-related genes and delayed endocytosis.

Zn(II)_2_Cys_6_-containing proteins are transcription factors that are unique to fungi and have a variety of regulatory functions that differ between fungi. Some reports have shown that the Zn(II)_2_Cys_6_ transcription factor is related to secondary metabolites: a Zn(II)_2_Cys_6_ transcription factor *sirZ* regulates the biosynthesis of the epipolythiodioxopiperazine (ETP) toxin, sirodesmin PL in *Leptosphaeria maculans* [27] and the Zn(II)_2_Cys_6_ transcription factor *rolP* regulates leucinostatin production in *Paecilomyces lilacinu* [28]. FpUme18 has a similar function in regulating the production of secondary metabolites of *F. pseudograminearum*. The absence of *FpUME18* decreased the production of DON toxin in *F. pseudograminearum*; this could also be one of the reasons for the decrease in virulence.

Zn(II)_2_Cys_6_ proteins have a GAL4 domain and usually have transcriptional activation activity [29]. In the methylotrophic yeast *Candida boidinii*, Trm1p was responsible for the transcriptional activation of several methanol-inducible promoters, and the deletion of *TRM1* completely inhibited its growth on methanol [30]. In *Aspergillus niger*, the Zn(II)_2_Cys_6_ transcriptional activator InuR was involved in the regulation of inulinolytic genes, and the deletion of the *inuR* gene was shown to result in a severe reduction in growth on inulin and the sucrose medium [31]. In *Aspergillus nidulans*, the Zn(II)_2_Cys_6_ transcriptional activator AmyR regulated the expression of amylolytic genes and was shown to preferentially localize to the nucleus in response to isomaltose [32]. In this study, we found that FpUme18 did not have a transcriptional activation activity through experiments in yeast; perhaps FpUme18 might need the assistance of other proteins or some modification of proteins in vivo to play a role in transcriptional activation, or it cannot perform functions in a heterogeneous system. The mechanism through which FpUme18 is involved in the regulation of gene expression in *F. pseudograminearum* needs to be further clarified. Endocytosis is a biological process in which extracellular substances are internalized into vesicles [33]. The development of the mycelia of filamentous fungi requires vesicles to transport nutrients [34]. The delayed endocytosis caused in *ΔFpume18* corresponded with the defects of the radial growth of mycelia and the slowed-down growth rate of colonies. When *FpUME18* was deleted, vesicle trafficking was blocked, which reduced the efficiency of substrate materials for spore germination and mycelial growth. *ΔFpume18* also showed sensitivity to H_2_O_2_ in vitro; this might make it difficult for *ΔFpume18* to degrade ROS that are produced by the host during pathogen infection. In addition, *ΔFpume18* was also sensitive to NaCl and CR. The defects of endocytosis and their response to external environmental pressures might be connected with the reduced pathogenicity of *F. pseudograminearum*.

To summarize, FpUme18 plays an important role in growth, conidial production, germination and endocytosis in *F. pseudograminearum* and also contributes to balanced salt stress, cell wall integrity, reactive oxygen resistance and full virulence. This work enriched the study of the mechanism of *F. pseudograminearum* infection in wheat and laid a foundation for the prevention and control of *Fusarium* crown rot.

## 4. Materials and Methods

### 4.1. Strains and Culture Conditions

The *F. pseudograminearum* wild-type strain WZ-8A was kept in tubes with glycerol in a freezer at −80 °C. All the fungal cultures were incubated on PDA (potato dextrous agar, 200 g potato, 20 g dextrose, 20 g agar in 1 L of distilled water) plates at 25 °C in darkness. Liquid YPG (3 g yeast extract, 10 g peptone, 20 g glucose in 1 L of distilled water) was used to prepare fungal mycelia for the extraction of DNA, RNA and proteins. For fungal conidiation, fresh culture blocks were transferred from PDA plates into 150 mL flasks containing 100 mL of CMC (1.5% Carboxymethylcellulose) in a medium with shaking at 150 rpm and 25 °C for 4 days.

### 4.2. FpUME18 Gene Deletion and Complementation

The *ΔFpume18* mutant was generated with the split-marker approach [35]. A 983 bp upstream flanking sequence and a 1260 bp downstream flanking sequence of the *FpUME18* gene were amplified from *F. pseudograminearum* genomic DNA using a PCR. The partial fragments of the hygromycin phosphotransferase gene (*HPH*) were amplified from the vector pKov21. These three fragments were linked by an overlapping PCR. Two fusion fragments containing the flanking of *FpUME18* and part of *HPH* were amplified using corresponding primer pairs (Figure S1A). The purified fragments were mixed and transformed into WZ-8A protoplasts and mediated by polyethylene-glycol (PEG). For complementation, the full length of the *FpUME18* gene with its native promoter regions-eliminating-stop codon was amplified and inserted into the pYIP102 vector cut by restriction endonuclease *Hind* III (New England Biolabs, Houston, Texas, USA) following the instruction of the one-step cloning kit (Vazyme C112, Vazyme, Nanjing, China), generating pYIP102-*FpUME18*-3FLAG with the confirmation of sequencing. This construct was used for the protoplast transformation of the *ΔFpume18* mutant. After G418 screening, PCR and Western blot validation, the complement transformants that were similar to the wild type were selected.

### 4.3. Protein Extraction and Western Blot Assays

The three mycelium blocks were inoculated in a 250 mL conical flask with a YPG liquid medium and cultured with shaking at 150 rpm and 25 °C for 3 days. Mycelia were collected by filtering them through a miracloth in the funnel, followed by rinsing with sterile double distilled water and a precooled PBS (phosphate-buffered saline) buffer. The mycelia were packed with paper towels that removed extra liquid. The mycelia were frozen in liquid nitrogen and ground into a fine powder using a pestle and mortar before being dissolved in PBS containing 1 mM of PMSF (Phenylmethanesulfonyl Fluoride) (Beyotime ST506, Beyotime, ShangHai, China). Total proteins were separated on a 12% SDS-PAGE gel, and the FpUme18-flag fusion protein was detected specifically using a primary anti-FLAG antibody (1:10,000 dilution, Abmart M20008, Abmart, ShangHai, China). The primary antibody was tested using a secondary antibody anti-Mouse (1:10,000 dilution, Abmart M21003S). The signal in the blot was visualized with ECL kits (Abbkine BMU102-CN, Abbkine, Wuhan, China) and photographed with a GE Image Scanner (General Electric Company, Boston, Massachusetts, USA).

### 4.4. Vegetative Growth Assays

The strain was incubated in a 25 °C incubator for 3 days in darkness, and a 5 mm plug was taken from the edge of the colony. The mycelial plug was placed upside down in the center of a 9 cm Petri dish containing 15 mL of a PDA medium. It was sealed and placed in a 25 °C incubator for 3 days in darkness. The colony diameter was measured using the cross-over method, and the average colony diameter was calculated. The experiment was conducted in three biological repeats, with three replicates each time.

### 4.5. Conidiation and Conidia Germination Assays

For the test of conidiation, the mycelium blocks were cultured in a 150 mL flask with 50 mL of a CMC liquid medium at 25 °C with 150 rpm for 5 days. The conidia were harvested by filtering through sterile a miracloth, followed by washing three times with sterile distilled water. They were then counted on the hemocytometer under a microscope. For conidia germination, the conidia suspension at the concentration of 5 × 10^5^ conidia/mL was dropped on a sterile glass slide with a pipet, placed in a Petri dish, and put into an incubator at 25 °C in darkness. After 8 h, the conidia were counted under a microscope and photographed. All experiments were conducted in three biological repeats, with three replicates each time.

### 4.6. Stress Sensitivity Assays In Vitro

Fresh mycelial plugs, 5 mm in diameter, were cultured on PDA plates or supplemented with 1.5 M sorbitol, 1 M NaCl, 0.05% sodium dodecyl sulfate, 0.2 g/L of Congo red or 10 mM H_2_O_2_ at 25 °C in darkness for 3 days. The colony diameter was measured, and the growth inhibition rates under different stress conditions were calculated. Experiments were conducted in three biological repeats, with three replicates each time.

### 4.7. FM4-64 Staining

To prepare the stock solution, 10 μL of 8 mM FM4-64 (Sigma Cat. S6689, Sigma Aldrich, St. Louis, MO, USA) was transferred via a pipet into a sterile microcentrifuge tube containing 90 μL of DMSO. The fresh conidia were washed three times with sterile distilled water, resuspended in 99 μL of distilled water and supplied with 1 μL of an FM4-64 stock solution. The spores were observed and photographed under a Zeiss microscope at different staining times (Carl Zeiss, Oberkochen, Germany). For the mycelia sample, fresh fungal blocks were put into a 250 mL flask containing 100 mL of the YPG medium for incubation with shaking at 25 °C and 150 rpm. After 14 h of incubation, the mycelia were harvested and rinsed with sterile water, and diluted in an FM4-64 stock solution. After staining, the mycelia were washed with sterile water and observed on the glass slide under a Zeiss microscope and photographed at 900 ms for the exposure time.

### 4.8. Pathogenicity Assays

The seeds of the susceptible wheat variety Aikang 58 were surface disinfected in 75% alcohol for 30 s and rinsed with sterile distilled water. They were left to grow in the soil for 3 days after germination. The seedlings were then carefully pulled out and put in a tray, and the roots were moisturized with absorbent paper. A fresh mycelium block, 5 mm in diameter, was inoculated on the wheat coleoptile. The mycelium blocks were removed after 24 h of dark treatment, and the lesions were measured after 3 days of inoculation. Experiments were conducted in three biological repeats, with three replicates each time. At 24 hpi, the inner epidermis of the wheat coleoptile was peeled off and observed under a Zeiss microscope.

### 4.9. DON Toxin Production Determination

The wheat grains were washed and soaked overnight, then boiled in boiling water for 30 min. After a day, 70 g were bottled and autoclaved for 1 h. Each conical bottle was inoculated with four mycelium plugs, with three replicates per group. The treatment was dark at 25 °C for 30 days, during which time it was shaken regularly. The content of DON was determined using high-performance liquid chromatography. The experiment was set up with three replicates, and three samples were taken from each replicate.

### 4.10. Transcriptional Activation Analysis in Yeast

The *FpUME18* fragment with the homologous arm of a pGBKT7 vector using *F. pseudograminearum* cDNA as a template was amplified and inserted into the pGBKT7 vector and was cut by restriction endonuclease *Sma* I (New England Biolabs, Houston, Texas, USA), following the instruction of a one-step cloning kit (Vazyme C112). The reporter plasmid pGBKT7-*FpUME18* and the negative control plasmid pGBKT7 were transformed into the yeast strain AH109 individually. The transformed yeast strains were cultured on SD/-Trp and SD/-Trp-His-Ade + X-α-gal media at 30 °C.

### 4.11. Quantitative PCR Assay

In order to analyze the expression of *FpUME18* during infection, fresh mycelia were collected and inoculated into the wheat coleoptile, and the mycelia were collected at different time points. To analyze the expression of phospholipid-related genes, the mycelium blocks were cultured in a CMC liquid medium at 25 °C with 150 rpm for 4 days before the spores were transferred to a YPG medium in a flask and cultured at 25 °C and 150 rpm for 12 h. Germinated spores were collected using a sterile filter cloth. To analyze the expression of trichothecene biosynthesis genes, the wheat coleoptile was infected with the mycelium block for 48 h, and the outer layer of the wheat coleoptile was collected. The total RNA was extracted from the collected samples using a Trizol reagent (Vazyme R401-01) according to the manufacturer’s instructions and reverse-transcribed into cDNA with HiScript III RT SuperMix (Vazyme R323-01). Quantitative expression assays were performed using an SYBR green (Vazyme Q711-02) kit in an ABI Real-time PCR (Applied Biosystems, San Francisco, CA, USA) detection system. *EF1α* was used as the internal standard reference gene for normalization. The primers for real-time PCR are listed in Table S1. The 2^−ΔΔCt^ method was used to calculate the relative expression level. The expression of *FpUME18* and phospholipid-related genes was measured once, and three replicates were set up, while the expression of trichothecene biosynthesis genes was detected three times, with three replicates each time.

## Data Availability

Data are contained within the article or Supplementary Material.

## Supplementary Materials

The supporting information can be downloaded at: https://www.mdpi.com/article/10.3390/ijms241310987/s1.
