# Supplementary material for: A Putative Zn(II)2Cys6-Type Transcription Factor FpUme18 Is Required for Development, Conidiation, Cell Wall Integrity, Endocytosis and Full Virulence in Fusarium pseudograminearum
Source: Int J Mol Sci. 2023 Jul 1;24(13):10987. doi: 10.3390/ijms241310987 (PMC10341630; doi:10.3390/ijms241310987)
Supplement: Supplementary file 1 [file ijms-24-10987-s001.zip › ijms-2415251-supplementary.pdf]

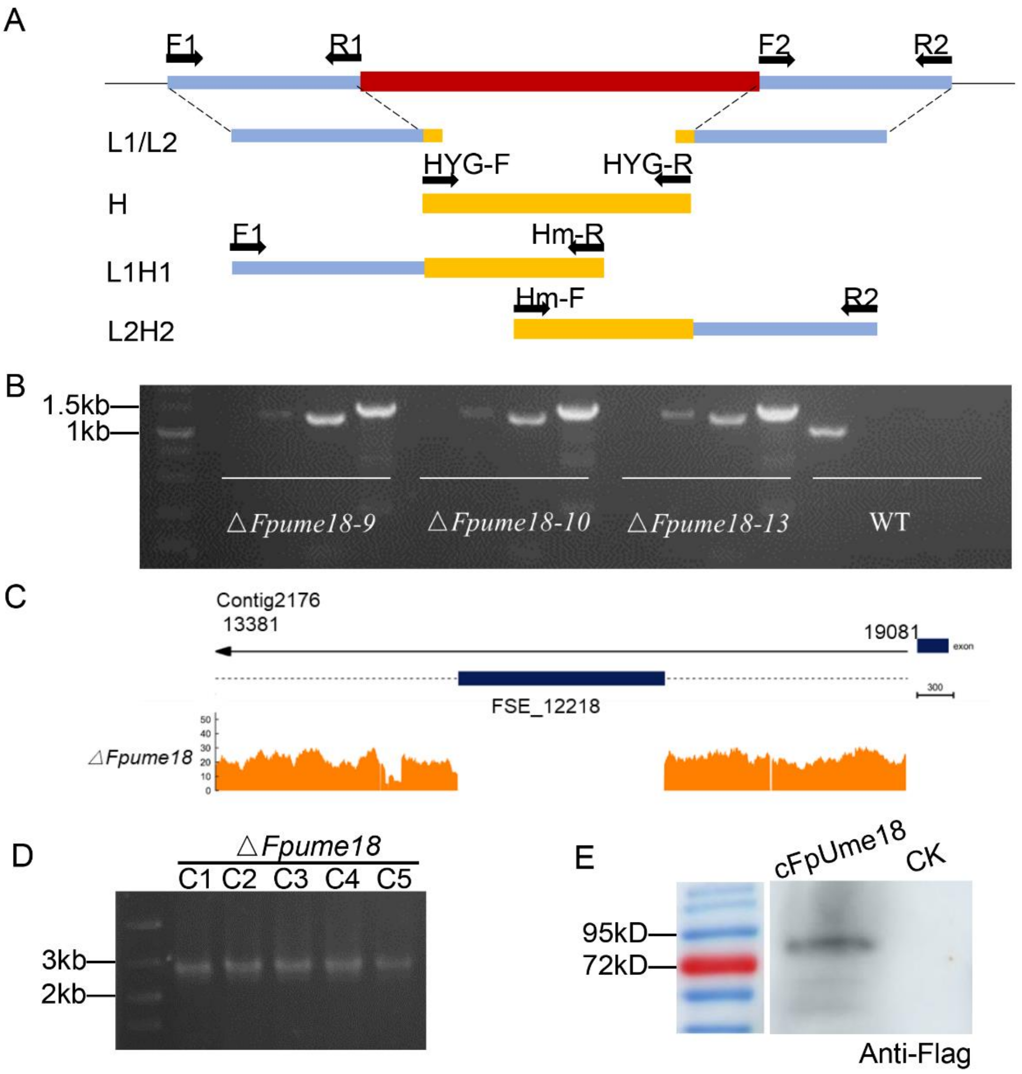

Figure. S1 Targeted gene replacement of *FpUME18* and genetic complementation. (**A**) Schematic graph for gene knockout strategy of *FpUME18*. (**B)** Confirmation of *FpUME18* deletion mutants by PCR strategy. Four pairs of primers were used to detect *FpUME18* gene; the positive PCR was applied to test the fragments of upstream, downstream and hygromycin cassette and the sizes of fragment length is 983bp, 1280bp, 1240bp, and 1380bp, respectively. **(C)** Resequencing result of *FpUME18* deletion strain. (**D)** PCR verification for FpUme18 complementary strain and the amplified *FpUME18* gene and promoter fragment was 2759 bp. (**E)** Western blot verification of FpUme18-flag fusion protein of complementary strain.

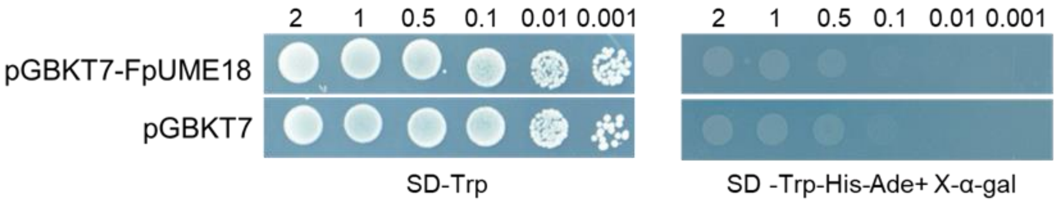

Figure. S2 Transcriptional activation activity analysis of FpUme18 protein in yeast. The vector pGBKT7-FpUme18 and control vectors pGBKT7 were separately transformed into the yeast strain AH109. The transformants were grown on selective dropout/–tryptophan (SD/-Trp) and selective dropout/-tryptophan-histidine-adenine (SD/-Trp-His-Ade) medium at 30°C for 3 days.

Table S1 Primers for experiments

Primer	Sequence
FpUme18-F	AAGAGGAACGTGAAACGTCGG
FpUme18-R	ATGTATGCTGTCCGATCCTCG
EF1α-F	GAGGCTGGTATCTCCAAGGAT
EF1α-R	TGATGGCAACAATGAGGTTC
FpUME18-1F	CAGACGGGGAAGACTGATCTG
FpUME218-1R	CCTCCACTAGCTCCAGCCAAGCCCCTACCACTTTTGTGCCGGTC
FpUME218-2F	GAGTAGATGCCGACCGCGGGTTCTAACATCATAATGGAATAGCCATT
FpUME218-2R	GCTCGAAAAGAGAGATAGCCTC
FpUME18-yF1	CGGTGATGATACGAGCAACATA
FpUME18-yF2	AGACTGAGAAGCGACGCCTTG
FpUME18-yR2	ACCTCGAGAGATTTGAAATGTCG
FpUME18-yR3	ATGGTAGAAGTGAGTCAGGGAT
FpUME18-pYIP102-F	GTCGACGGTATCGATAAGCTTTCAAAAGAGGTTCCCTTCACGTC
FpUME18-pYIP102-R	CAGGAATTCGATATCAAGCTTTACTAGCAGGGCTGGATATCCTA
FpUME18-BD-F	ATGGAGGCCGAATTCCCGATGAAGACCTCAATCTGCTGG
FpUME18-BD-R	TGCAGGTCGACGGATCCCCTATACTAGCAGGGCTGGATA
CHO1-RT-F	GGCTCTAACGGCAACACCAAGG
CHO1-RT-R	GCGAGGAGAAGATGGACATGACAC

CHO2-RT-F	GCCATGACCTTGAGCCATCAG
CHO2-RT-R	GCTGAGAAACCATATCATGGGT
INO1-RT-F	CACCTACACGGATGCGGAGATTC
INO1-RT-R	CCGACCAACATCATACCGACCTTG
INO4-RT-F	TGCATGATCAGATTGCAGAGCG
INO4-RT-R	GGTTCGAAAATTGTTGAGCTTGC
OPI3-RT-F	TTGGCGACTTGGCATTAC
OPI3-RT-R	GGCTTACCGTACCACAGA
TRI4-RT-F	GTTCGCGACGGATTCTT
TRI4-RT-R	TCTCATCTCGCATCTGGA
TRI5-RT-F	GCATGAACGGTTTGGGTC
TRI5-RT-R	ATGAGATCGTTGACCCAA
TRI6-RT-F	GTGCTCGCCATGAATCTA
TRI6-RT-R	GCCTGTAGTGATCTCGCA
TRI8-RT-F	TCTGTTCCGGATTATGAG
TRI8-RT-R	CGTGTTGATCGTGTTGAA

Table S2 Gene ID

Gene	NCBI ID
CHO1	FPSE_01124
CHO2	FPSE_04957
INO1	FPSE_03117
INO4	FPSE_01269
OPI3	FPSE_06024
TRI4	FPSE_12162
TRI5	FPSE_12160
TRI6	FPSE_12161
TRI8	FPSE_12164
